# The unexpected role of bioaerosols in the Oxidative Potential of PM

**DOI:** 10.1038/s41598-017-11178-0

**Published:** 2017-09-08

**Authors:** A. Samake, G. Uzu, J. M. F. Martins, A. Calas, E. Vince, S. Parat, J. L. Jaffrezo

**Affiliations:** 1 Université Grenoble Alpes, CNRS, IRD, IGE (UMR 5001), F-38000 Grenoble, France; 2 Air et Bio, F-73 000 Chambéry, France

## Abstract

Bioaerosols represent up to 15–25% of PM by mass, but there is currently no assessment of their impact on Oxidative Potential (OP), or capacity of particulate matter (PM) to produce damaging oxidative reactions in the human lungs. Here, the OP of selected bioaerosols (bacteria cells *vs* fungal spores) was assessed through the cell-free DTT assay. Results show that bioaerosols induce Reactive Oxygen Species (ROS) production, varying along the microorganism type, species, and concentration. Fungal spores show up to 10 times more ROS generation than bacterial cells. At the highest concentrations, fungal spores present as much oxidative reactivity as the most redox-active airborne chemicals (Copper, Naphtoquinone). Moreover, bioaerosols substantially influence OP of ambient PM and that of its chemical constituents: in presence of *A*. *fumigatus* spores, the OP of Cu/NQ is increased by a factor of 2 to 5, whereas, 10^4^ and 10^5^ *S*. *epidermidis* bacterial cells.mL^−1^ halves the OP of Cu/NQ. Finally, viable and gamma-rays-killed model bioaerosols present similar oxidative reactivity, suggesting a metabolism-independent cellular mechanism. These results reveal the importance of bioaerosols for PM reactivity. PM toxicity can be modified due to bioaerosols contribution or by their ability to modulate the OP of toxic chemicals present in PM.

## Introduction

Atmospheric particulate matter (PM) is defined as a complex and dynamic mixture of particles from both chemical and biological origins^1, 2^. Exposure to PM has been linked with a wide range of deleterious health effects in both humans and animals. These include among others, cardiopulmonary disease, lung cancer, or asthma^3–5^. Atmospheric concentration of PM is regulated (e.g. annual average threshold in Europe is 40 µg.m^−3^ for PM_10_), but more than mass, detrimental effects are strongly associated with PM composition, surface area and size^5–8^. The atmospheric research community is currently working on alternatives to the mass concentration of PM to find relevant health and exposure metric, involving more PM properties^9, 10^. One underlying mechanism explaining many health effects of PM is its capacity to carry out or catalyze the formation of reactive oxygen species (ROS) within lung cells, responsible for further oxidative stress and airway inflammation^9–12^. This intrinsic property of PM called oxidative potential (OP) is easily revealed in biochemical and acellular assays by monitoring anti-oxidants depletion when in contact with PM.

While the physical and chemical properties of PM have been extensively investigated, relatively little is known about primary biological airborne particles (PBAP) also known as bioaerosols^2, 13–15^. They comprise living and dead microorganisms such as bacteria, fungi, viruses, bacterial and fungal spores, and microbial fragments, endotoxins, mycotoxins, pollens etc.^13, 14, 16^. Microorganisms in bioaerosols can survive as single cells or attached to dust particles or hydrated aerosol particles. Most bioaerosols range in size from micrometer to submicrometer^17–19^. Due to their light weight and size range, bioaerosols can be transported over long distances^19^, for example, transferred by wind, making them complex, highly variable and ubiquitous in both indoor and outdoor environments. Cultivable bioaerosols (bacteria, fungi) have been detected at high concentration in indoor and outdoor air^20–23^. Recent findings indicate that in most areas, bioaerosols can account for up to 15–25% of total PM mass^13, 14, 16, 24^ and higher concentrations have been reported in Amazonian areas (from 74 to 80% of PM mass)^16, 24^. Their concentration and composition fluctuate primarily depending on the sources impacting the site under consideration (e.g. soil, lakes, sewage treatment plants, agricultural activities etc.), the aerosolization mechanisms, and the physical and environmental conditions (e.g. shape, size, temperature, relative humidity, etc.) prevailing at each site^19, 25^. Usually, airborne fungi exist as spores while bacteria are cells^26^. Bioaerosol concentration in indoor/outdoor air ranges from 10^1^ to 10^5^ microorganisms.m^−3^ ^20, 22, 27^. Higher concentrations (up to 10^5^–10^7^ microorganisms.m^−3^) have been measured in rainforest environments or polluted areas (e.g. waste water treatment plants, composting facilities etc.)^20, 28^.

More recently, attention has been given to the PM_10_ bioaerosol fraction since evidence is growing about their implication in negative effects of PM both on climate (e.g. bioaerosols can interact with UV radiation, photo-oxidants and can also serve as nuclei for ice crystals and cloud droplets, thereby influencing the formation of clouds and precipitation, etc.)^14–16^ and on human health^4, 14, 29^. Inhalation is the primary route of exposure to bioaerosols^30^. Inhalation of high concentrations of fungal spores, bacterial cells, and their derivatives in indoor/outdoor environments is associated with serious inflammation-related health risks such as asthma, chronic obstructive pulmonary disease, aspergillosis, and immunological reactions^3, 4, 26^. Bioaerosols can also be responsible for infectious diseases such as tuberculosis, meningitis, and legionellosis^22, 25^. In addition, dose-response relationships exist between inhalation of airborne microorganisms and the development or exacerbation of asthma symptoms in humans^31, 32^. As for the chemical components of PM, it is worth noting that the pathogenicity of bioaerosols also depends on the number of inhaled microorganisms, their chemical composition, and their size^30, 33^.

Studies addressing the OP of PM are currently focusing only on the chemical fraction^2, 3, 10, 34^, while the impact of bioaerosols are unaccounted for. For example, Charrier and Anastasio (2015) attributed about 30% of overall OP of PM to unidentified components of submicron fine particles collected in Fresno during summer^12^. So, a reasonable hypothesis could be that bioaerosols may account for part of this unexplained fraction. In support of this, Vaitlingtom *et al*., (2013) among others have shown biodegradation of hydrogen peroxide (H_2_O_2_) by cloud-borne microorganisms^35, 36^, highlighting that cloud-borne microbial communities could also interact with H_2_O_2_ oxidants (or other kinds of ROS) involved in the oxidative stress.

Therefore, our study was conducted in order to initiate the assessment the contribution of bioaerosols to the oxidative potential (OP) of ambient PM. Within this framework, measurement of OP with the acellular DTT assay^10, 34^ was firstly adapted to viable bioaerosols and reliability of the results was evaluated with ascorbic acid (AA) and the 2′,7′-dichlorofluorescin diacetate (DCFH) cell-free methods. Then, OP of isolated model bioaerosols (cells and spores) and that of model bioaerosols associated with real ambient PM and highly toxic model airborne chemicals (copper and 1.4-naphtoquinone) were assessed based on the acellular DTT assay, using realistic environmental concentrations of both bioaerosols and ambient or model PM. Linearity as a function of concentration of PM (i.e. chemical and biological fraction of PM) was also investigated. The final purpose of this work was to begin to explore some of the main processes by which bioaerosols may contribute to the overall OP of PM.

## Results and Discussion

### Oxidative potential of model bioaerosols

**Figure 1:**
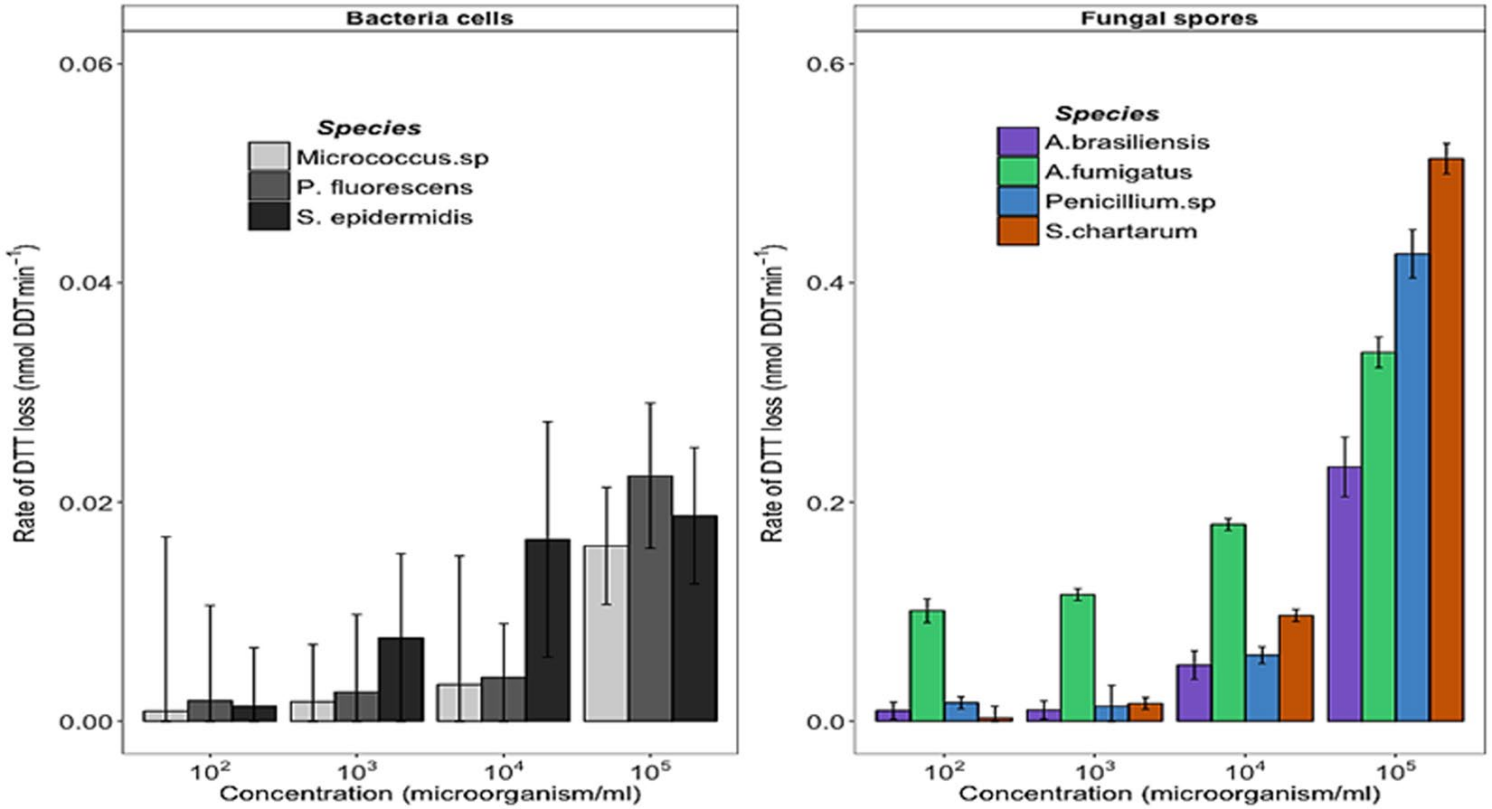
Intrinsic oxidative reactivity of model bacterial and fungal bioaerosols at increasing concentrations measured with the DTT assay. Error bars correspond to standard deviations calculated with triplicates. The highest bioaerosols concentration of 10^4^–10^5^ cells.ml^−1^ translated into atmospheric concentrations are commonly observed in occupational and polluted environments, while the lowest (10^3^ or less) are usually observed in clean outdoor atmospheres.

All of the tested model bioaerosols induced DTT depletion, which varied among the tested microorganisms and concentrations (Fig. 1). Likewise model bioaerosols, the oxidative reactivity of airborne redox-active chemical compounds (e.g. Cu, NQ, etc.) also varied with the tested species and concentration^12, 37, 38^. Bacterial cells induced a very low DTT oxidation (~10–20 pmolDTT.min^−1^) within the 30 min. experiments, but this rate tended to increase with increasing concentrations. Figure 1 also shows that fungal spores present ten times more oxidative reactivity than bacterial cells. Such ROS producing activity trends of model bioaerosols were also observed with DCFH acellular method (Fig. S1A). By contrast, the tested model bioaerosols were not able to induce significant ascorbic acid (AA) depletion (Fig. S1B). Such result with AA assay is not surprising since AA is a natural antioxidant that is usually used to assess oxidative potential of transition metals. In this assay, AA usually reduces metal ions and oxygen to finally generate hydroxyl radical, via a Fenton like reaction.

Among the tested fungal spores, only those of *A*. *fumigatus* presented a significant oxidative reactivity for all tested concentrations, consistently with the results of several *in vivo* and *in vitro* studies assessing the inflammatory potential of non-pathogenic fungal spores involved in airway inflammation^39, 40^. Shahan *et al*., (1998) demonstrated that the secretion of pro-inflammatory proteins (*e*.*g*. MIP-2, TNF-α etc.) by rat alveolar macrophages is differentially induced by fungal spores^39^. For instance, in this last study, among the tested *Aspergillus* species, the spores of *A*. *fumigatus* were able to stimulate the production of pro-inflammatory cytokines (MIP-2, TNF-α, KC etc.) while those of *A*. *terreus* were not. Other studies assessing the toxicological impacts of bioaerosols collected in various occupational backgrounds indicated that all sampled bioaerosols (*i*.*e*. *A*. *fumigatus*, actinomycetes, endotoxins, total bacteria etc.) are able to increase the production of ROS of the “granulocyte-like HL60” cell lines^41, 42^. The intensity of ROS production was dependent upon the nature and amount of bioaerosols^41, 42^. Indeed, Timm *et al*., (2009) demonstrated through a multiple regression modeling that the inflammatory potential induced by fungal spores better explains the total inflammatory potential of bioaerosols^41^, thus supporting the significant differences in antioxidant depletion observed between the bacterial cells and fungal spore tested in our study.

These findings indicate that inhalation of current indoor/outdoor and/or occupational environmental concentrations of bioaerosols (Table 1) could be responsible for respiratory impairments observed elsewhere^3, 4, 25^. To the best of our knowledge, there is currently no regulated exposure limit for bioaerosols although several recommended maximum limits have been proposed^4, 23, 40, 43, 44^. For instance, based on inflammatory respiratory effects in animal models, *Eduard et al*.,^4, 40^ have proposed a “lowest observed effect level” (LOEL) of 10^5^ spores.m^−3^ for non-pathogenic and non-mycotoxin producing fungal species. When converted to liquid concentrations (under the assumption of Table S1), this LOEL is in the same range of that of fungal spores that presented significant oxidative reactivity in the present study. However, one should keep in view that the DTT assay is a cell-free method which therefore does not take into account cellular and individual susceptibility (i.e. of animals or humans). Studies on bacterial and fungal aerosols in diverse central and eastern European indoor environments have proposed the values of 1 to 7.10^3^ viable and cultivable bacterial cells.m^−3^ as a limit for dwellings and communal premises^40, 43, 44^. For such non-pathogenic bacteria concentrations, the oxidative reactivity measured in this study was always insignificant. Hence the use of this low threshold should ensure a better protection against airborne non-pathogenic bacterial cells for potentially exposed persons.

**Table 1:** Concentrations of viable and cultivable bioaerosols in different environmental backgrounds and conversion rates of tested concentrations (adapted from^18, 22, 23, 27^).

Tested concentrations (Bacteria or spores.ml^−1^)	Tested concentrations, converted in (Bacteria or spores.m^−3^)	Indoor air (Bacteria or spores.m^−3^)	Outdoor air (Bacteria or spores.m^−3^)	Occupational settings (Bacteria or spores.m^−3^)
10^5^	1,82^*^10^5^	10^1^–10^4^	10^1^–10^5^	10^1^–10^7^
10^4^	1,82^*^10^4^			
10^3^	1,82^*^10^3^			
10^2^	1,82^*^10^2^			

^*^Occupational settings correspond to composting facilities.

### Effect of bacterial cells on the intrinsic oxidative reactivity of model atmospheric chemicals

**Figure 2:**
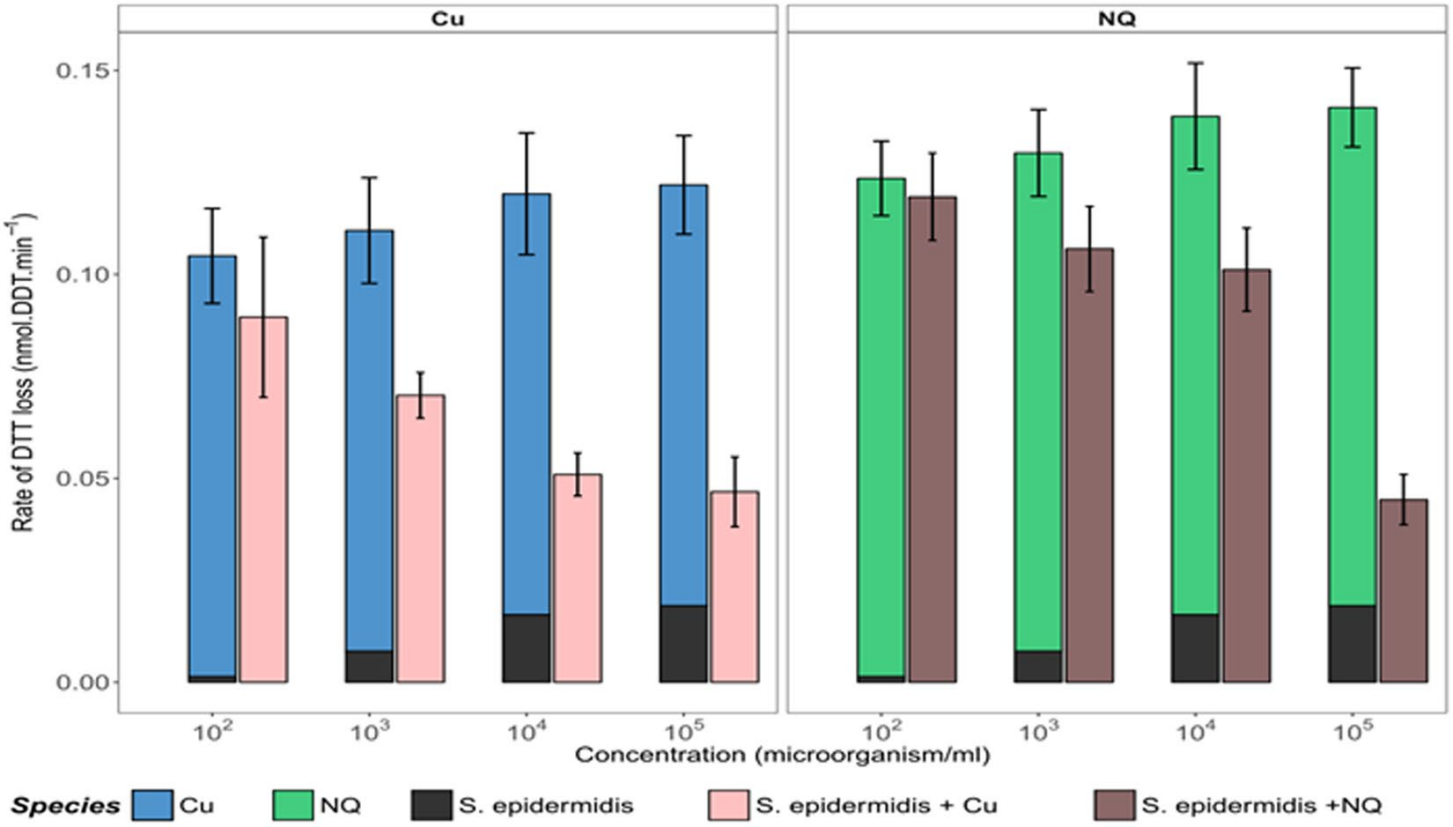
Oxidative reactivity of copper (Cu) and 1,4-naphthoquinone (NQ) in the presence of increasing concentrations of *S*. *epidermidis* cells, measured with the DTT cell-free assay. Error bars correspond to standard deviations calculated with triplicates. Rates of DTT loss from single species and mixtures of *S*. *epidermidis* cells and Cu/or NQ are compared to those calculated as the sums of individual solutions. Asterisks indicate cases where measured and calculated rates of DTT loss are statistically different (Wilcoxon rank-sum test, n = 3, p ≤ 0.1).

To evaluate if the presence of bioaerosols significantly influences the oxidative reactivity of model airborne chemical compounds, we measured the oxidative reactivity of 1 µM copper (Cu) and 1 µM naphtoquinone (NQ) in absence or presence of *S*. *epidermidis* cells at increasing concentrations (S. *epidermidis* was chosen because it presented a clear dose-response relationship when in contact with DTT, as shown in Fig. 1). As illustrated in Fig. 2, the oxidative reactivity of Cu gradually decreased when the concentrations of *S*. *epidermidis* cells increased. The oxidative reactivity of Cu was halved when in contact with 10^4^ to 10^5^ bacterial cells.mL^−1^. At concentrations equal or lower than 10^2^ cells.mL^−1^, no effect was observed on the OP of Cu. Similar trends were observed with NQ, indicating that *S*. *epidermidis* cells are able to modulate the intrinsic reactivity of Cu and NQ towards DTT. This clearly suggests that this bacterial species presents an “anti-oxidant” effect against both Cu and NQ.

This protective effect may be explained by the interaction of these chemicals (adsorption or uptake) with bacterial cells, thus reducing their availability for reacting with DTT. Numerous studies have shown that bacterial cells walls present huge amounts of reactive sites (carboxyl, phosphoryl, amino, or sulfate groups) capable of chemical complexation^45–48^. Once deprotonated, these groups become anionic and therefore available for complexation of cationic chemicals (such as Cu^2+^) in aqueous solution. The processes involved in the limitation of quinones’ oxidative potential could be different, as quinones can diffuse freely into the lipid bilayers of the cells walls, where they can be trapped^49–51^, thus becoming less available for reacting with DTT. Moreover, some studies have evidenced the formation of hydrogen bonds between the reactive functional groups present on bacterial cells walls and quinones or derivatives^49^. These two types of interactions between *S*. *epidermidis* cells walls and NQ or Cu may explain the lower reactivity of NQ (or Cu) in presence of important concentrations of *S*. *epidermidis* cells.

The same experiment was performed with *P*. *fluorescens* and *Micrococcus sp*. but only at a concentration of 10^5^ cells.mL^−1^ (because of their low DTT response at lower concentrations, Fig. S2). The observed trends were slightly different from those measured with *S*. *epidermidis* cells. These bacterial species presented low oxidative reactivity, which added up to that of Cu (additive depletion of DTT). However this was not the case for NQ, for which intrinsic oxidative reactivity decreased in the presence of the bacterial cells. For example, in the presence of *Micrococcus sp*., the oxidative reactivity declined by almost 40% with the mixture as compared to that calculated as an individual sum from NQ and *Micrococcus sp*. The discrepancy in effects of the 3 tested bacterial species on oxidative reactivity of Cu or NQ could be due to differences in cell wall composition (different nature and density of functional groups). The cell wall of Gram positive bacteria (e.g. *S*. *epidermidis*, *Micrococcus sp*., etc.) is known to contain important amounts of peptidoglycan (rich in reactive carboxyl, phosphoryl and amino groups etc.) as well as of teichoic and teichuronic acids (containing carboxyl groups)^45, 46^. On the contrary, Gram negative bacterial cells (e.g. *P*. *fluorescens*) present external membranes with many proteins (glycosylated proteins, hydrophilic channels or porins etc.), much less peptidoglycan and lack teichoic and teichuronic acids^45–47^, thereby being potentially less reactive towards Cu or NQ.

### Effect of fungal spores on the intrinsic oxidative reactivity of (atmospheric) model chemical compounds

**Figure 3:**
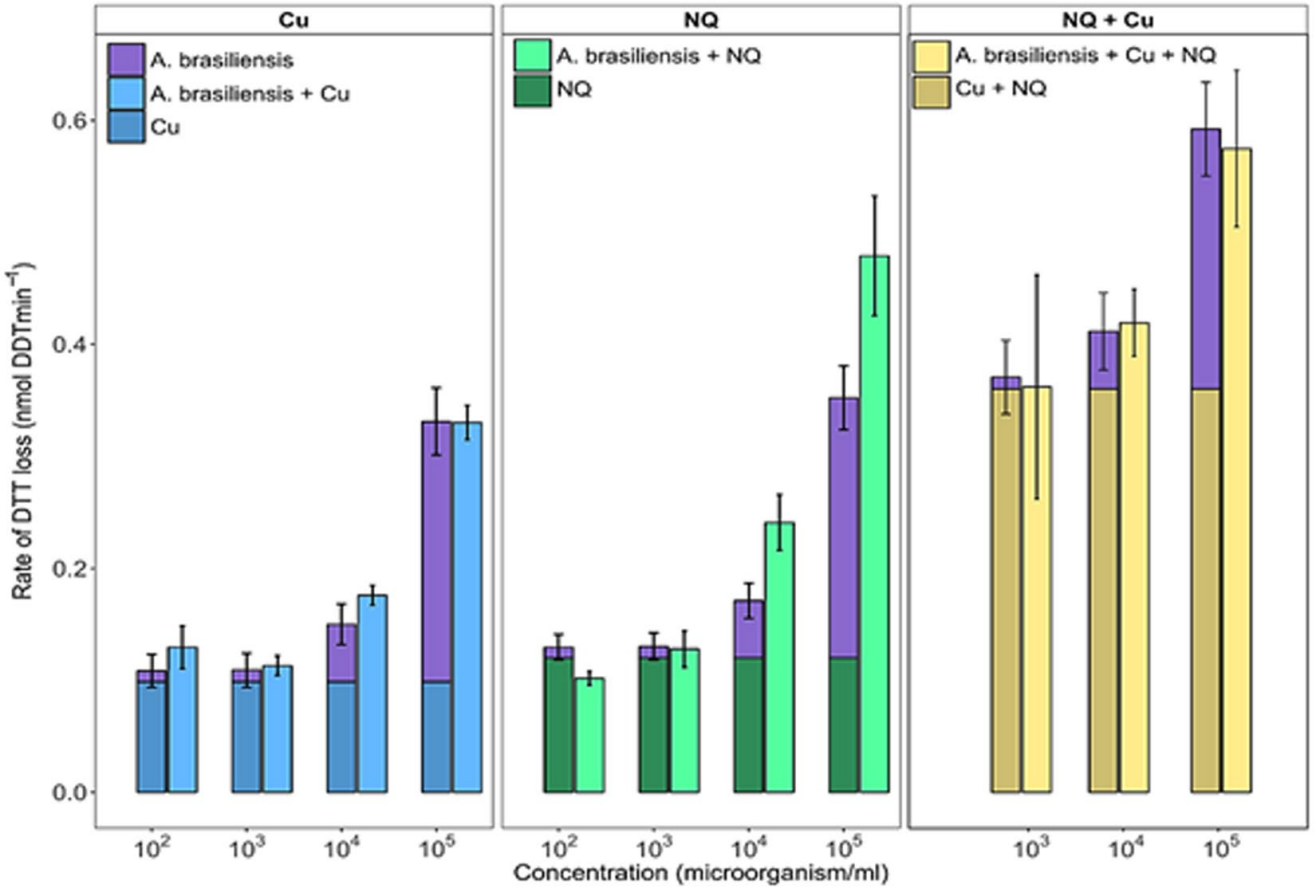
Comparison of the rates of DTT depletion induced by mixtures of *A*. *brasiliensis* spores and model airborne redox-active chemicals (Cu, NQ and Cu + NQ) with those calculated as the sum of individual DTT depletion rates (clustered bar graphs). Statistical differences are highlighted with an asterisk (n = 3, p-value ≤ 0.1). Error bars represent standard deviations calculated from triplicate measurements.

Using the same procedure as for bacteria, the OP of 1 µM Cu and 1 µM NQ, (chemicals commonly observed in the atmosphere) was assessed in the presence of increasing concentrations of airborne spores of *A*. *brasiliensis*, *A*. *fumigatus*, *S*. *chartarum* and *Penicillium sp*. The results observed for *A*. *brasiliensis* are shown in Fig. 3 where the rate of DTT loss clearly varied with the concentration of fungal spores. For instance, the oxidative reactivity of Cu and NQ doubled and tripled in presence of 10^4^ and 10^5^ spores.mL^−1^ of *A*. *brasiliensis*, respectively. Similar increases in oxidative reactivity, but of variable intensity, were observed with the other fungal spores (see Fig. S3). These observations support the cumulative effect of the intrinsic oxidative reactivity of airborne chemicals and fungal spores.

To further investigate this cumulative effect of OP, we compared the rate of DTT depletion induced by a mixture of *A*. *brasiliensis* spores and Cu, NQ, or both chemicals, with the sum of DTT depletions measured with individual biological and chemical species used at the same concentrations. The results show good agreement between the DTT depletion measurements obtained with both methods (Fig. 3), confirming that the intrinsic oxidative reactivity of fungal spores and airborne chemical compounds can be considered as cumulative. These observations are in agreement with the results of studies showing linearity and additivity of individual contributions of airborne chemical species to overall OP^12, 37^.

### Effect of cell viability on the oxidative reactivity of atmospheric PM

Since both viable and dead microorganisms can be found in atmospheric PM, in parallel to the reactivity of viable airborne microorganisms, we also studied their oxidative potential after gamma-rays inactivation. It is worth noting such physical inactivation method of living cells/spores was chosen in order to avoid the use of toxic chemicals, which are likely to interfere with the acellular assay. Gamma-rays do not alter cell-wall integrity in the short term unlike other methods used to inactivate microorganisms such as thermal or chemical treatments. Optical microscopy pictures of inactivated cells and spores are provided in Figure S4. The intrinsic oxidative reactivity of inactivated fungal spores and bacterial cells was evaluated in the presence and absence of real ambient PM samples. This approach also gave information on the main process involved in ROS generation by bioaerosols.

**Figure 4:**
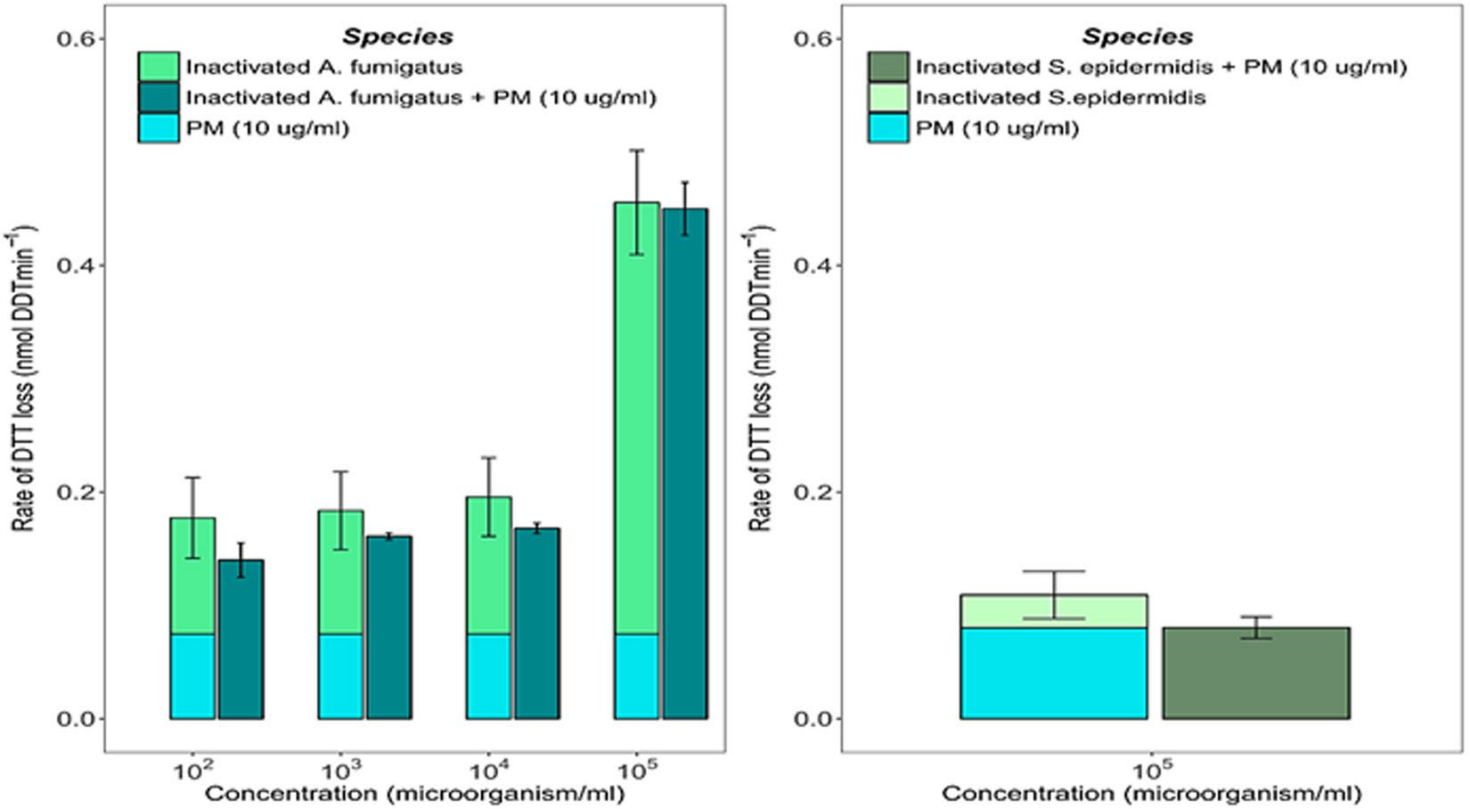
(I) Rates of DTT consumption by model bioaerosols inactivated by gamma-rays: (**a**) = Spores of *A*. *fumigatus* and (**b**) = *S*. *epidermidis* cells. (II) Clustered bar graphs highlight the rate of DTT loss induced by the mixture of model bioaerosols and ambient PM collected in Passy (Vallée de l′Arve, France) whereas the stacked bars were calculated as the sum of individual oxidative reactivity of ambient PM and gamma-rays inactivated airborne microorganisms. Error bars correspond to standard deviations calculated with triplicates.

The results presented in Fig. 4 show that inactivated spores of *A*. *fumigatus* present the same DTT response as viable spores. Similarly, the rate of DTT loss induced by the mixture of inactivated spores of *A*. *fumigatus* and ambient PM collected at the urban background site of Passy (Vallée de l′Arve, France) remained equal to the sum of individual reactivity. Similar patterns were also obtained with ambient PM collected in a very different environment which is a subway platform in Toulouse, France, (Fig. S5). This suggests that the contributions of inactivated fungal spores and ambient PM add up, as already shown with viable cells. Similar patterns can be observed in Fig. 4 with *S*. *epidermidis* inactivated cells, although the effect is less pronounced. These findings highlight that oxidative reactivity of airborne microorganisms is most probably based on a non-active mechanism since dead spores/and cells present quantifiable OP values similar to those measured with active cells and spores (Fig. S6). These results indicate that the oxidative process could relate to the outer cell/spore wall reactivity. It could be hypothesized that intrinsic oxidative reactivity of airborne microorganisms derive from passive redox-activity induced by the high density of functional chemical groups present at the external part of the wall of all living/dead cells, and in particular of bacterial cells and fungal spores, which could ensure DTT dehydrogenation into its disulfide form in presence of dioxygen, one of the major reactions involved in ROS generation through the DTT cell-free assay (chemical mechanisms are provided in Fig. S7).

Several *in vivo* studies are also related to this question, but they are not yet fully conclusive. Our results are consistent with the work by Hohl et *al*., (2005), based on the *in vivo* study of immune competent mice, which demonstrated that *A*. *fumigatus* spores induce comparable inflammatory cell influx before and after being heat-killed^52^. This last study also showed that active metabolism is not critical for the underlying process, which is probably mediated by β-glucans exposed at the outer spore-walls^52^. Other findings suggested more complicated processes implying an “unknown” protein^53, 54^. For instance, by comparing control groups with mice inoculated with viable and non-viable *P*. *chrysogenum* spores, Cooley et *al*., (2000) indicated that mice exposed to viable spores produce significant total IgE serum whereas that exposed to non-viable spores did not^53^. This suggests that the process is not related to the spore/cell wall constituents, because the same constituents are found onto both active and non-viable microorganism wall. Noteworthy is that the precise underlying mechanism is still not clearly understood^55^.

## Conclusions

This study aimed at evaluating the intrinsic oxidative reactivity of model bioaerosols (bacterial cells and fungal spores) through the use of an acellular assay (DTT depletion), and their role in the overall Oxidative Potential of atmospheric PM. Four main results were obtained. (1) The tested model bioaerosols presented intrinsic oxidative reactivity, which varies with the type of microorganisms (bacterial cells *vs* fungal spores), the species, and the cells/spores concentrations. At the highest tested concentrations of 10^4^ and 10^5^ spores.mL^−1^ (which could translate into the current atmospheric levels of bioaerosols observed in specific occupational backgrounds such as composting facilities, agricultural zones etc.), fungal spores present oxidative reactivity similar to those of the most redox-active airborne chemicals (*e*.*g*. Cu and NQ) and up to 10 times more reactivity than bacterial cells. At concentrations equal or lower than 10^3^ microorganisms.mL^−1^ (which could translate into the most current indoor/outdoor bioaerosol levels), all of the tested model bioaerosol presented insignificant OP, except *A*. *fumigatus* which is an opportunistic pathogens. (2) The OP of mixtures of fungal spores with natural PM or model airborne chemicals (Cu or NQ) are similar to the sum of individually measured OP values, thus confirming a cumulative effect of the OP of mixed reactants. (3) Bacterial cells (*S*. *epidermidis*) strongly reduced the OP of Cu and NQ, in relation with a protective action of these bacteria against oxidation, probably related to cell wall chelation of these chemicals, which thereby decreases their availability for reacting with DDT. This mechanism has been widely considered for modeling interactions between bacterial cells and heavy metals, referring to a surface complexation process. (4) Finally, experiments with gamma-rays inactivated airborne microorganisms have shown that oxidative mechanisms occurring at the surface of fungal spores and bacterial cells are probably non-metabolic (passive) as viable and gamma-rays inactivated cells or spores presented similar OP values. Although further studies are needed to better understand the underlying mechanisms, these results suggest that ROS production by bioaerosols is probably independent of cell′s metabolism.

Altogether these results show the importance of accounting for the contribution of bioaerosols when assessing the overall oxidative potential of ambient PM. Further work is needed to better understand the role of bioaerosols in the global toxicity of PM especially by considering the complexity of airborne microbial communities (bacteria cells, fungal spores, viruses etc.) and their byproducts (endotoxins, mycotoxins, etc.), pollen, plants debris etc. which can positively or negatively modulate the OP of PM. It would be important to fully assess the impact of bioaerosols on health for the initiation of health alerts by authorities, since both the type of airborne microorganisms and their concentration were shown in the present study to control the oxidative reactivity of natural PM.

## Material and Methods

### Selection of model bioaerosols

Some common atmospheric bacterial and fungal species have been selected^17, 18, 23, 30, 56, 57^. For bacteria, two Gram positive cocci (*Staphylococcus epidermidis* and *Micrococcus sp*.*)* and one Gram negative rod (*Pseudomonas fluorescens)* were selected. Concerning fungi, spores of *Stachybotrys chartarum* and *Penicillium sp*. and of *Aspergillus brasiliensis* and *Aspergillus fumigatus* were used. These fungal species are commonly used in exposure models because of their frequent occurrence in indoor air contamination^55^.

*A*. *fumigatus* is involved in various health issues such as severe invasive aspergillosis in immune-compromised people, or in allergic bronchopulmonary aspergillosis. *S*. *chartarum* is of particular interest in indoor environments due to its ability to colonize damps and to produce mycotoxins (trichotecenes) responsible for respiratory and neurological effects^22, 58^. It should be emphasized that all selected microorganisms are commonly found in outdoor or indoor environments.

### Sampling airborne bioaerosols

Commercial reference strains (CIP69.13 T, ATCC 14 990 and ATCC 16404, respectively) were used for *P*. *fluorescens*, *S*. *epidermidis* and *A*. *brasiliensis*. The other species were isolated from urban and natural environments: indoor for *Micrococcus sp*., *S*. *chartarum* and *Penicillium sp*., and outdoor for *A*. *fumigatus*. Air samples were collected using a one stage Andersen impactor at a flow rate of 28.3 L.min^−1^ for 4 minutes^59^. These microorganisms were directly impacted onto solid culture media: Malt Extract Agar culture for fungi and Trypticase Soy Agar for bacteria. Plates were incubated at 27 °C for 5 days. Colonies were identified by macroscopic and microscopic examinations, using lactophenol blue stain for fungi and Gram staining for bacteria.

### Culture of isolated model bioaerosols

For each previously isolated and identified microorganism species, a single colony was cultured on biological culture media: Luria-Bertani (LB, Biolife Italiana, Milan) for bacterial cells and Malt agar (MA, Biolife Italiana, Milan) for fungal spores. The inoculated culture plates were incubated at 30 °C, for 2 and 5 days, for bacterial cells and fungal spores, respectively. This step was intended to collect the cells and/or spores in their exponential growth phase (See Fig. S8A,B).

For bacterial cells, a single colony of each isolated strain was inoculated in 10 mL Luria Bertani liquid medium (LB, Biolife Italiana, Milan) and grown at 30 °C for 2 days, with agitation at 150 rpm. Preliminary experiments indicated that the liquid culture media were not suitable for the cell-free OP assays, due to very fast and important depletion of antioxidant in the negative controls. These media contain reactive chemical compounds, such as transition metals (e.g. Fe), which are known to cause high DTT depletion. To avoid such interferences during the DTT assay, bacterial cells suspensions were centrifuged for 10 min at 5000 g (at ambient temperature). The bacterial pellet was suspended aseptically in 10 mL of sterile chelex treated phosphate buffer ([PO_4_] = 1 M; pH = 7.4 ± 0.1). This cells-cleaning process was repeated three times.

Concerning fungal spores, a single colony of each isolated microorganism was inoculated onto malt agar plates and then grown at 30 °C for 5 days. The growth of fungal spores into liquid medium gave rise to filamentous structures (mycelia), which were not investigated in the present study. Hence, after fungi culture onto solid culture media for 5 days, fungal spores were collected in sterile ultrapure deionized water (UP Water), by gently scraping the surface of culture medium with sterile swabs. The swabs were soaked in 10 mL UP water and stripped by vortexing, in order to recover the maximum of spores. This process was performed several times to get enough spores without incorporating culture medium in spores’ suspensions.

### Quality and enumeration of microbial suspensions

To ensure that there was no contamination during the cells/spores culture steps, culture purity was visually controlled by optic microscopy (Axioscope, Zeiss): 10 µL of microbial suspensions and serial dilutions (1:10 to 1:10000) were loaded between glass slides and coverslips. The presence of single shape cells was verified systematically and the number of cells/ spores was determined in order to determine liquid concentrations: at least 100 bacterial cells or fungal spores were counted in several microscope fields^60^, in order to obtain enumerations with estimated errors lower than 15%.

### Gamma rays inactivation of bioaerosols

To evaluate if the oxidative potential of airborne microorganisms is linked with active or passive metabolic mechanisms, some model bioaerosols were inactivated with gamma-rays. Briefly, microbial suspensions prepared in glass tubes were irradiated with gamma-rays at 10 kGy for 16 h (CEA facilities, Grenoble, France). After irradiation, cell wall integrity was checked by optical microscopy and the absence of cell viability was also tested by culturing aliquots of the gamma-irradiated samples, which revealed always negative.

### Sampling ambient particulate matter (PM) - Extraction of atmospheric PM and pure chemical compounds

PM_10_ samples were collected on quartz filters with a high-volume air sampler (DA-80) at a flow rate of 30 m^3^/h over 24 h. Sampling was achieved in different environments in France: Passy (Vallée de l′Arve, in winter)^61^ and Toulouse (subway station).

To evaluate the effect of bioaerosols on the OP of ambient PM, 5 mm diameter punches of filters were extracted in UP water by shaking for 2 hours at 37 °C in the dark. Filter extracts were then centrifuged (at 1500 rpm for 5 min) at 4 °C, to separate the filter residue and the supernatant, which was used for the OP measurement.

For model chemical compounds, appropriate amount of copper (Cu) and 1,4-naphtoquinone (NQ) were also dissolved in UP water by shaking the for 40 min at 37 °C in the dark condition. These chemical species were selected since they are known to be highly redox-active and widely used as positive controls in OP studies^12, 34^.

### OP measurement: adaptation of the DTT assay to bioaerosols oxidative potential measurements

The dithiothreitol (DTT) assay is a cell-free method commonly used to assess the oxidative potential of airborne redox-active chemical compounds^62, 63^. Briefly, DTT consumption is continuously monitored when in contact with PM, and the depletion of DTT (in excess) is proportional to the concentration of redox-active species present in PM. The protocol established by Charrier and Anastasio, (2012) was adapted to fit our constraints, and significant changes were brought in the sample pretreatment step^10, 34^.

**Figure 5:**
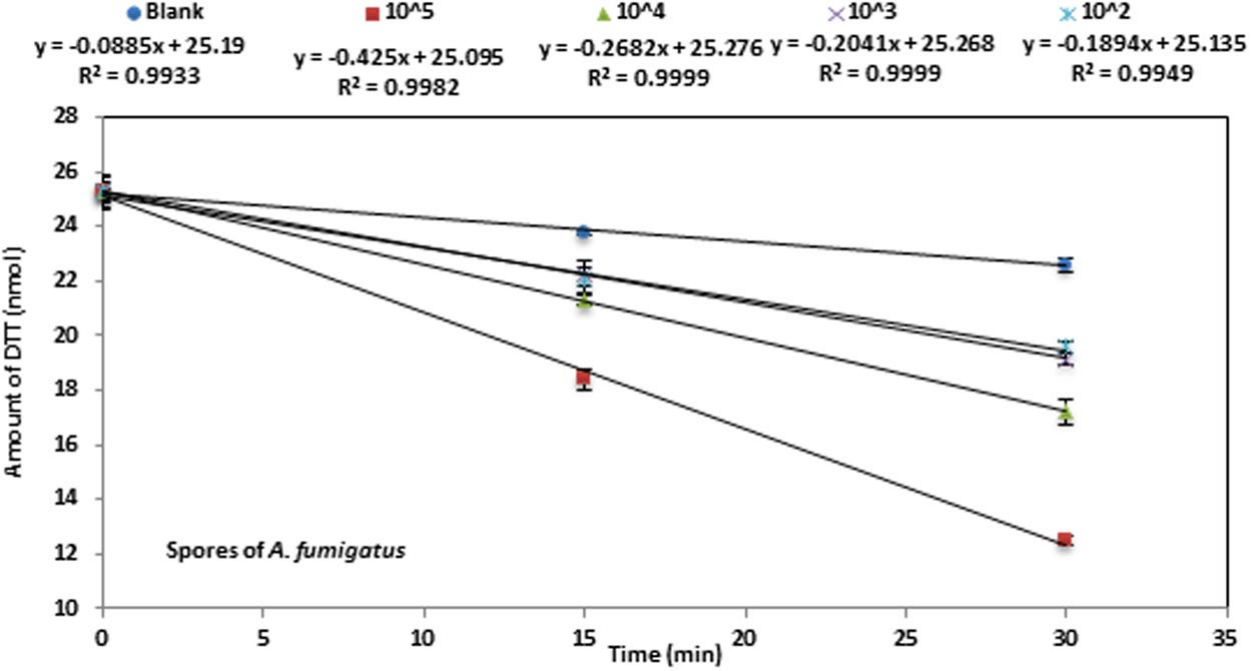
Kinetics of DTT consumption by the model bioaerosols (case of *A*. *fumigatus*). Each data point is the average of triplicate measurements. Error bars represent the standard deviation calculated with triplicates (when not visible, they are smaller than symbols).

The loss of DTT was monitored with a semi-automated method conducted in 48 wells plates: intrinsic light absorbance of the mixture was measured at 412 nm on a plate-reader (TECAN spectrophotometer Infinite® M200 pro). The procedure was as follows: 50 µL of model bioaerosols suspensions and/or 50 µL of PM extracts were incubated with an appropriate quantity of 1 M phosphate buffer (pH = 7.4 ± 0.1, pre-incubated at 37 °C) in a final volume of 550 µL. Triplicates of each sample were used for kinetic studies. The reaction was initiated by adding 50 µL of DTT (0.5 mM DTT solution in phosphate buffer) to each well (including the negative controls). The reaction was stopped at 3 specific times (0, 15 and 30 min) by adding 100 µL of 1 µM 5,5′-dithiobis (2-nitrobenzoic acid) (DTNB). The reaction between the remaining DTT and DTNB gives rise to a yellowish color (thionitrobenzoate; TNB) which can be monitored at 412 nm (maximum absorbance). Measurement repeatability was assessed by calculating the coefficient of variability (CV) between triplicates. The calculated CV were always lower than 5%. The rate of DTT loss, which indicates the ability of model bioaerosols to produce ROS, was determined from the slope of the linear regression of DTT remaining amount *vs* time, as shown in Fig. 5 (each time point was calculated as average ± standard deviation). The rate of DTT depletion was measured under conditions ensuring linearity of this rate, i.e. when less than 30% of initial DTT amount is depleted. The amount of remaining DTT was obtained from equation (1):

1 $${N}_{DTT}=\frac{{N}_{0}\times Ab{s}_{t}}{Ab{s}_{0}}$$

where N_DTT_ indicates the amount of DTT at time t (nmol), N_0_: is the amount of DTT (expressed in nmol) at time t = 0, Abs_0:_ is the absorbance corresponding to the initial amount and Abs_t_: is the absorbance measured at time t.

The intrinsic rate of DTT loss for all samples was calculated by subtracting blank values (or negative controls) from each sample value within the same experiment.

The rate of DTT depletion is assessed in presence of increasing realistic environmental concentrations of bioaerosols. Tested concentrations ranged from 10^2^ to 10^5^ microorganisms/ml. Such concentrations could correspond to the exposure levels currently observed in both indoor/or outdoor or/and occupational backgrounds (e.g. composting facilities). Table 1 indicates either the tested concentrations of model bioaerosols expressed in microorganisms.ml^−1^ and corresponding values in microorganisms per cubic meter of inhaled air. This conversion was performed with the following equation:

2 $$Nb/{m}^{3}=\frac{(Nb/mL)\times 20\,ml}{11\,{m}^{3}}$$

where Nb/m^3^: is the concentration of viable microorganisms in air (microorganisms.m^−3^); Nb/mL: is the concentration of microorganisms in liquids (microorganisms.ml^−1^); 20 ml is the estimated volume of lung fluid and 11m^3^ is the volume of inhaled air within a 24 h, by an adult human^30^. To simplify the calculation, we assumed that inhaled microorganisms remain into the lung fluid for 24 h, are viable and fully transferred to the deep lungs.

### Statistical analysis

Differences between the rate of DTT loss from single mixture of PM components (e.g. Cu, NQ, model bioaerosols) and those calculated as the sum of individual rates were analyzed with the non-parametric Wilcoxon rank-sum test. This non-parametric method was chosen because of the small sample size (n = 3). Statistical differences are indicated with asterisks (p ≤ 0.1).

## Electronic supplementary material

Supporting information
