# Supplementary material for: The unexpected role of bioaerosols in the Oxidative Potential of PM
Source: Sci Rep. 2017 Sep 8;7:10978. doi: 10.1038/s41598-017-11178-0 (PMC5591310; doi:10.1038/s41598-017-11178-0)
Supplement: Supplementary file 1 — Supporting information [file 41598_2017_11178_MOESM1_ESM.pdf]

Supporting information for: The unexpected role of bioaerosols in the Oxidative Potential of PM

A. Samake[1], G. Uzu[1*], J. M. F. Martins[1], A. Calas[1], E. Vince[1], S. Parat[2] and J. L. Jaffrezo[1]

[1] Univ. Grenoble Alpes, CNRS, IRD, IGE (UMR 5001), F-38000 Grenoble, France.
[2]Air et Bio, F-73 000 Chambéry, France

[*] Corresponding author: gaelle.uzu@ird.fr

This file contains 12 slides, including this one.

Figures S1;S2, S4, S6, S8 are additional graphics.
Figures S4, S9 are additional optical microscopic images.
Figure S7 is a pattern.
Figure S10 are microorganism viability tests.

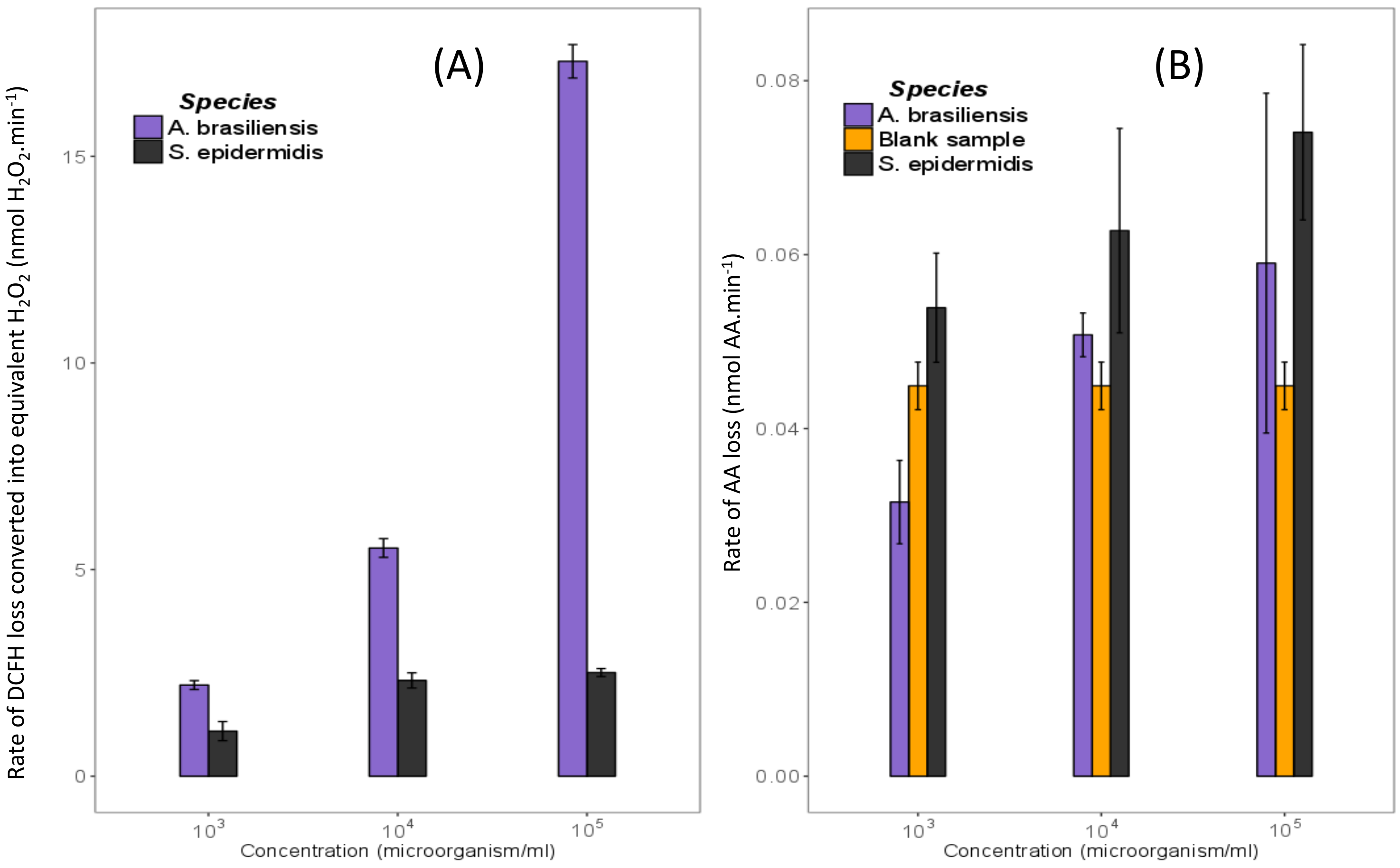

Figure S1: Intrinsic oxidative reactivity of *A. brasiliensis* spores and *S. epidermidis.* bacterial cells at increasing concentration, evaluated with: (A) Dichlorofluorescin diacetate (DCFH) and (B) Ascorbic acid cell free assays respectively. Error bars are standard deviation between triplicates of measurements.

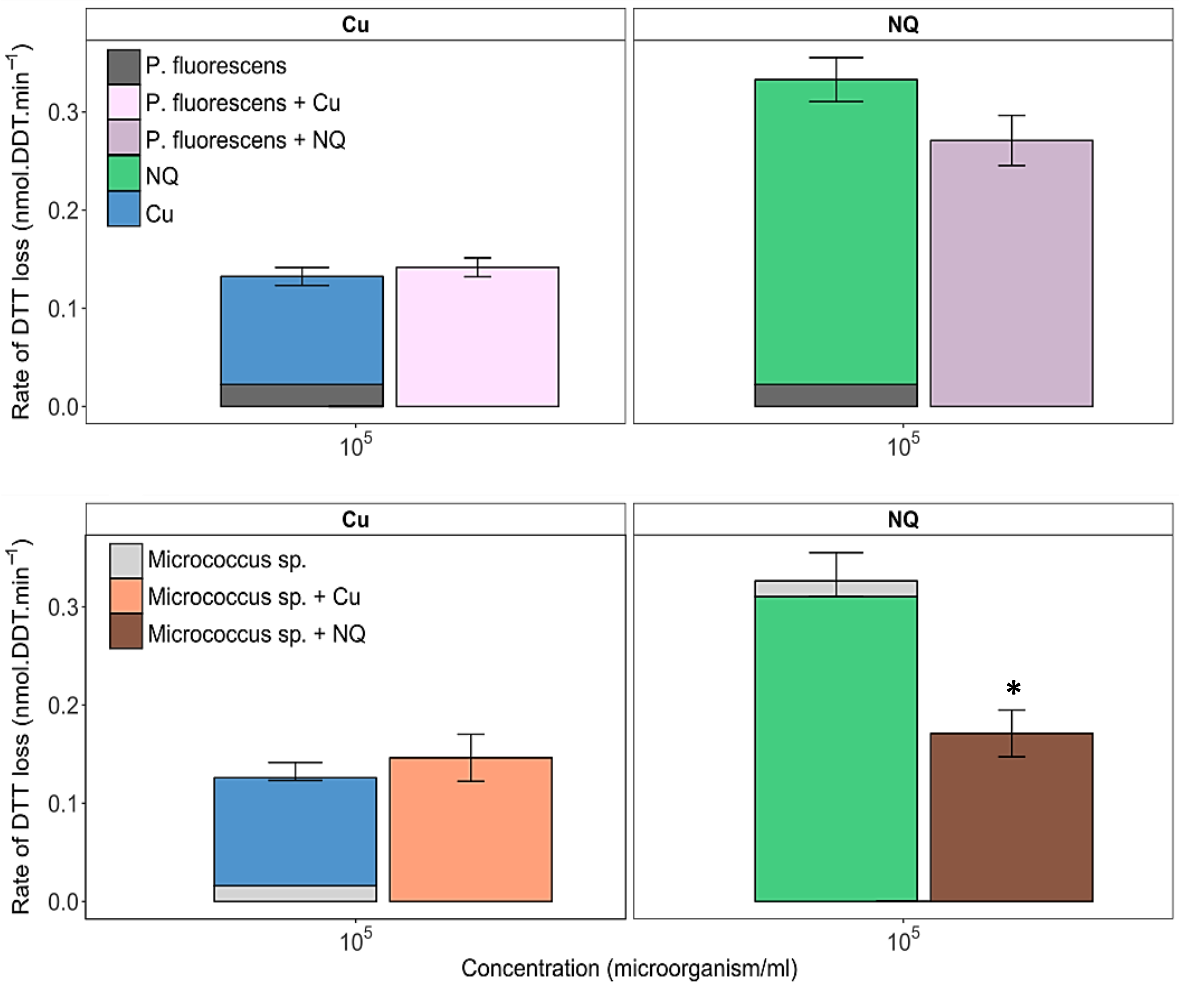

Figure S2: Oxidative reactivity of copper (Cu) and 1,4-naphthoquinone (NQ) in the presence of 10^5 *P. fluorescens*/or *Micrococcus sp.* bacterial cells, assessed with DTT cell-free assay. Error bars are standard deviation between triplicates of measurements. Asterisks indicate significant difference between the measured and the individual sum of DTT depletion rate (n=3, Wilcoxon rank-sum test, $p \leq 0.1$).

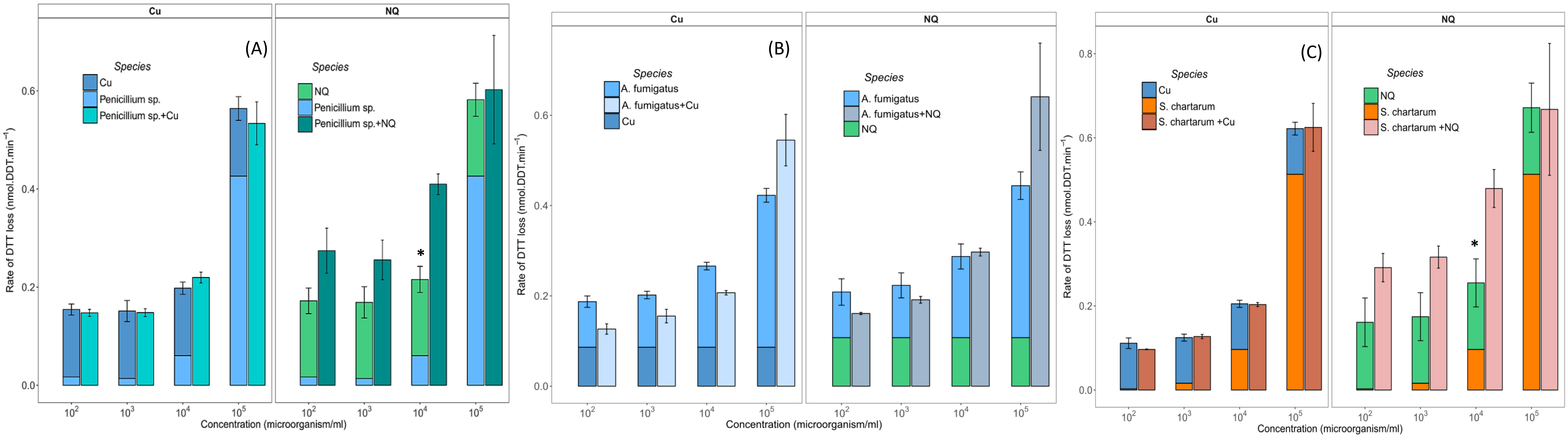

Figure S3: Evolution of intrinsic oxidative reactivity of copper and (Cu) and 1,4-naphtoquinone (NQ) in the presence of increasing concentrations of: (A) Spores of *Penicillium sp.,* (B) Spores of *A. fumigatus* and (C) Spores of *S. chartarum.* Oxidative reactivity has been evaluated with DTT cell-free assay. Error bars indicate standard deviation between triplicates of measurements. Asterisks underline cases where the measured and that calculated as the sum of individual rate of DTT loss are significantly different (n=3, Wilcoxon rank-sum test, p≤ 0.1).

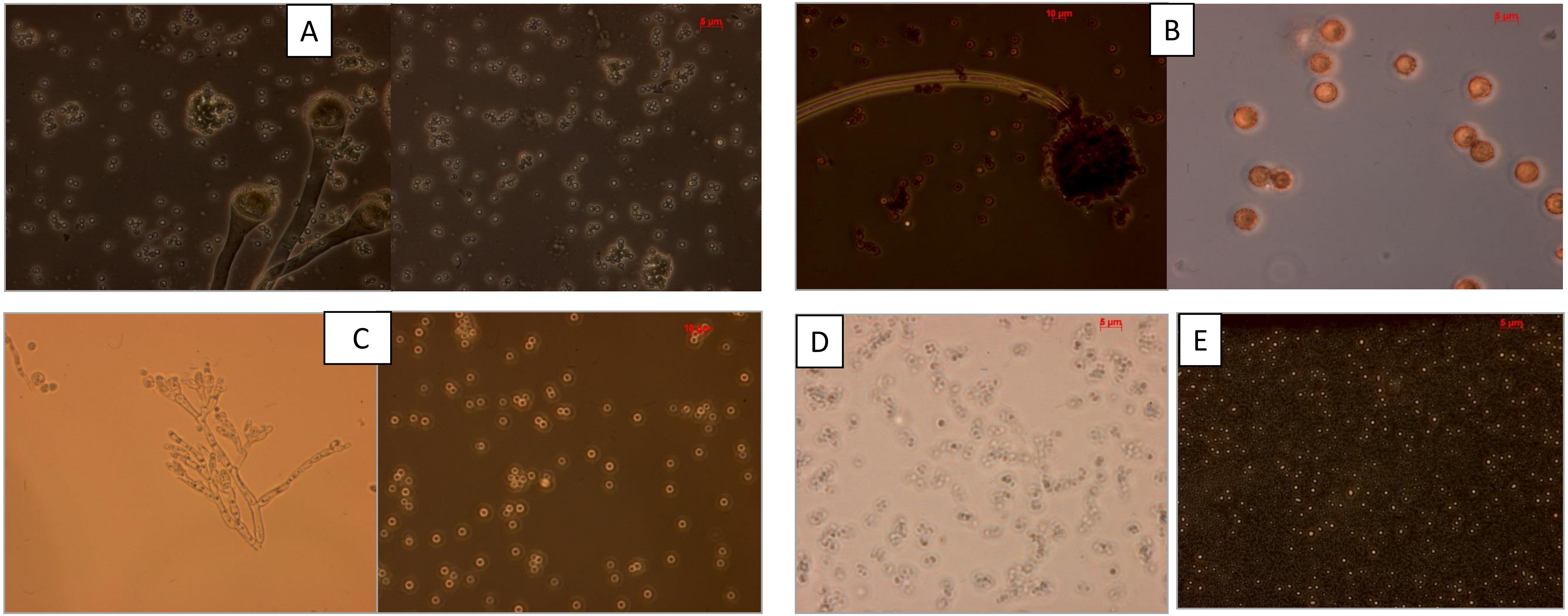

Figure S4: Optical microscopic images of gamma-rays inactivated microorganisms: (A)= *A. fumigatus*, (B)= *A. brasiliensis*, (C)= *Penicillium sp.;* (D)= *Microccus sp.;* (E)= *S. epidermidis,*

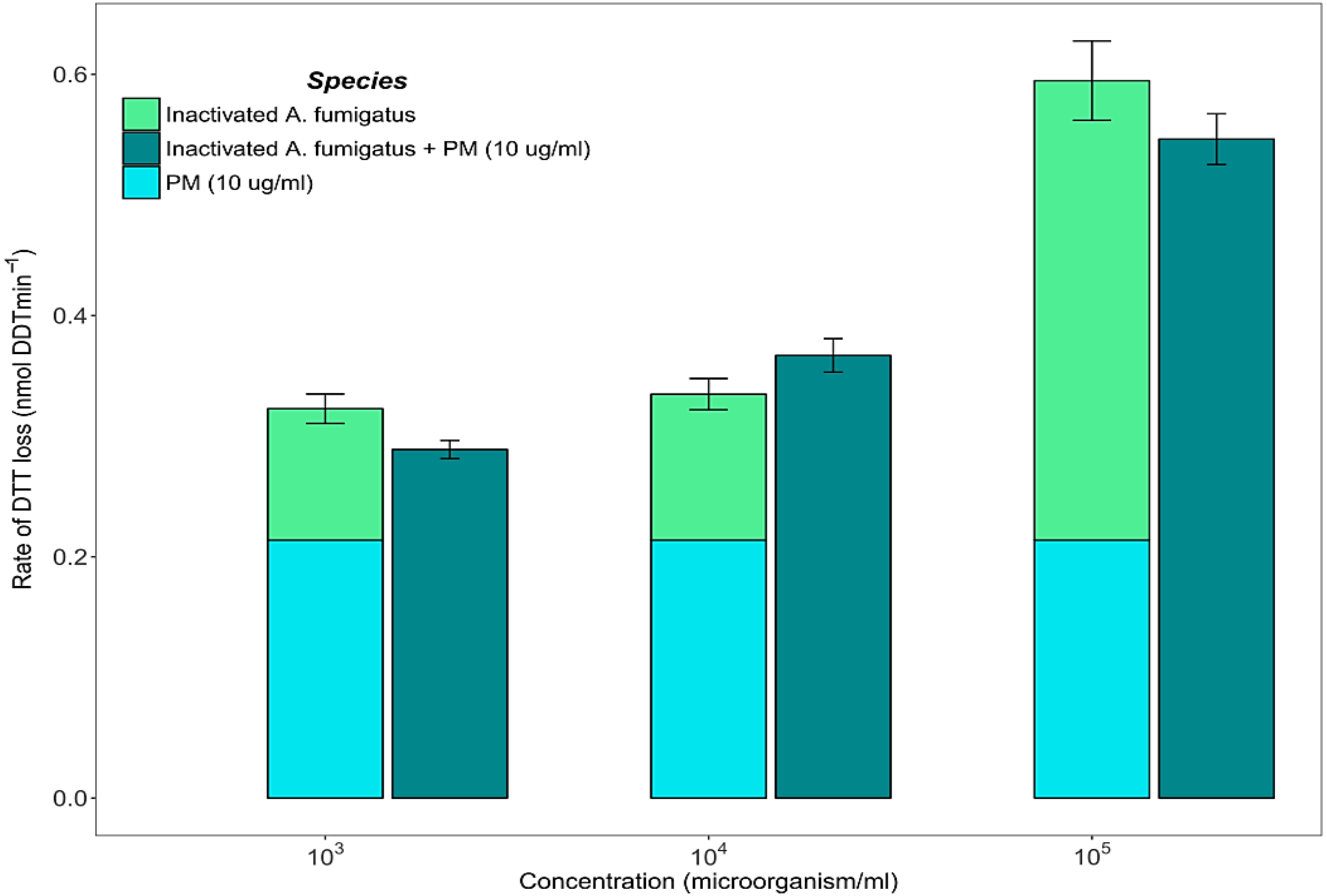

Figure S5: Rate of DTT depletion by a single mixture of gamma-rays inactivated *A. fumigatus* spores and ambient PM collected in Toulouse (subway station) compared to that calculated as sum of individual oxidative reactivity. Statistical differences between measured and calculated rate of DTT loss were analyzed with Wilcoxon rank-sum test (n=3, p≤ 0.1). Error bars are standard deviation between triplicates of measurements.

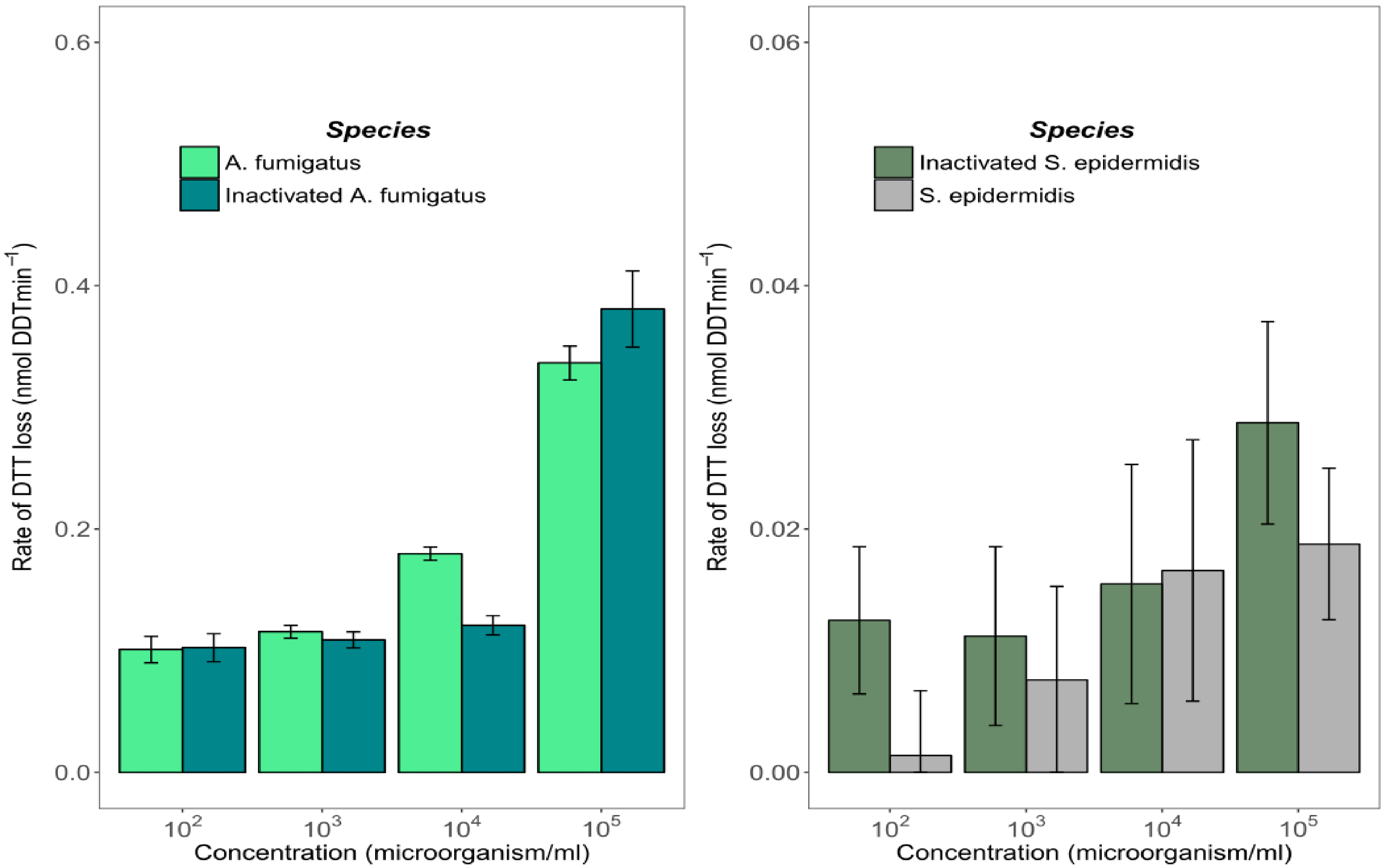

Figure S6: Rate of DTT depletion from viable microorganisms compared to that from gamma-rays inactivated ones. Statistical difference are underlined with asterisks different (n=3, Wilcoxon rank-sum test, p≤ 0.1Error bars are standard deviation between triplicates of measurements.

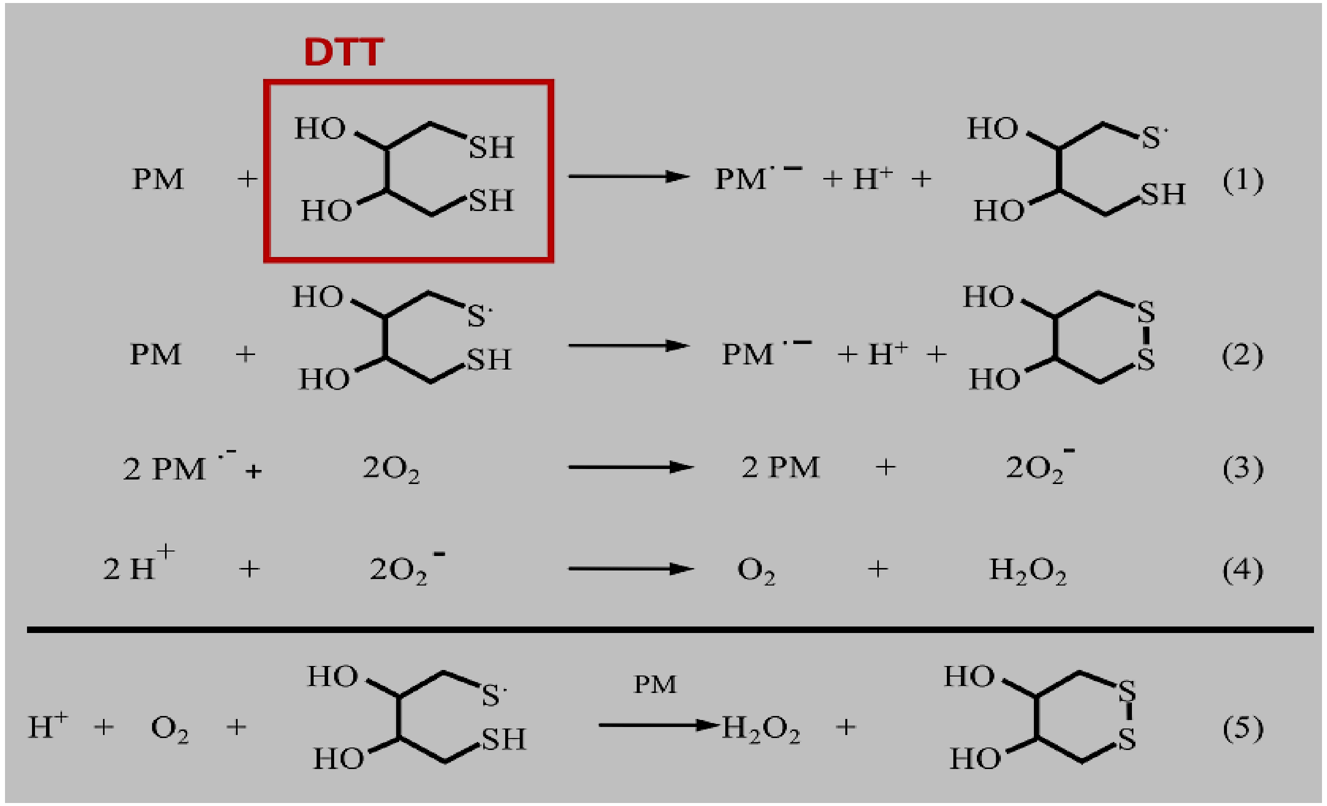

Figure S7: Chemical basis of DTT oxidation by redox-active contents of PM (adapted from Rattanavaraha et al., 2011 and therein references)

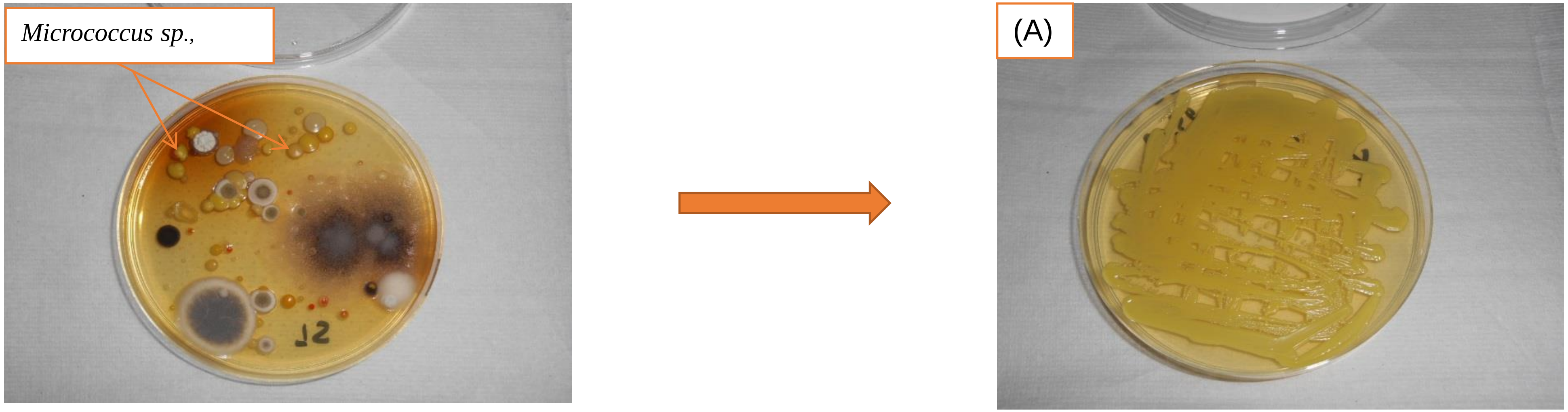

Figure S8A: Growth of bacteria cells collected onto Trypticase soy agar culture medium and specific subculture of identified *Micrococcus sp*., bacterial cells onto Luria-Bertani agar culture medium (A).

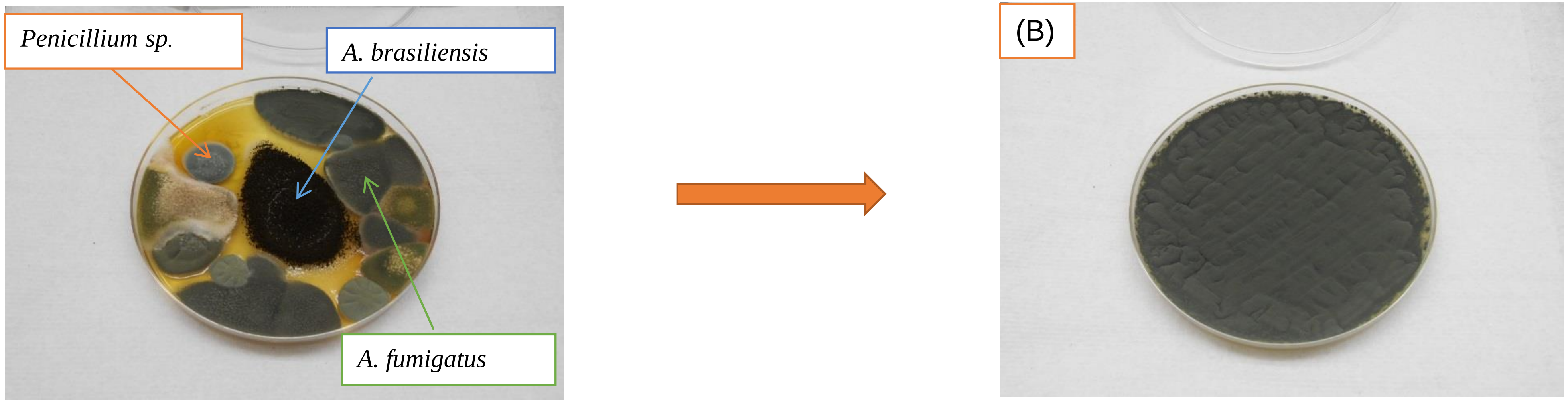

Figure S 8B: Growth of Fungal spores collected onto Malt Agar culture medium (MA) and specific subculture of identified *A. fumigatus* spores onto MA (B).

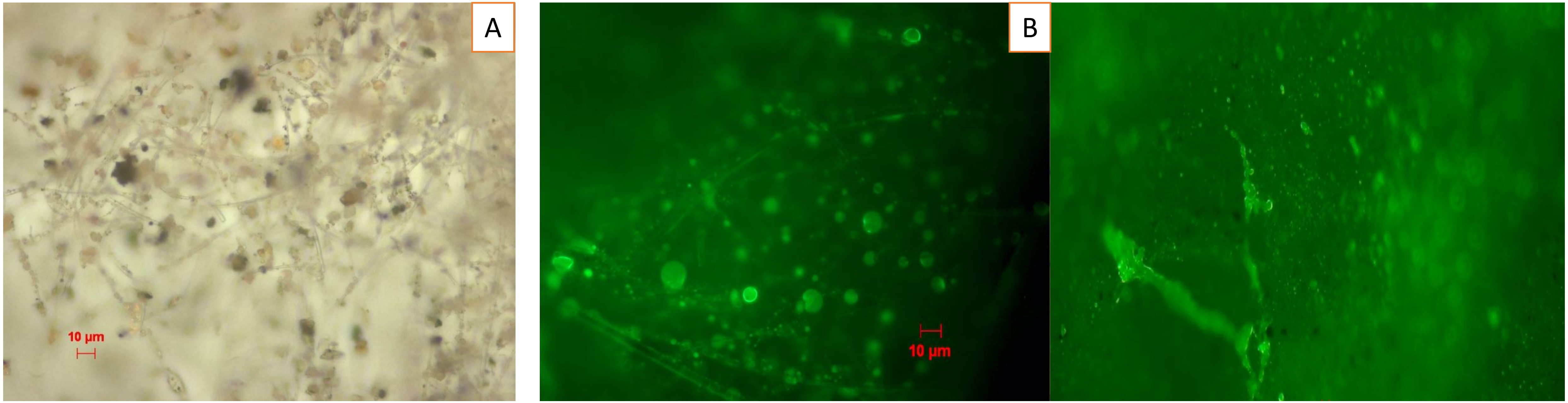

Figure S9: Optical microscopic examination of ambient particulate matter (PM) collected in Passy (Vallée de l'Arve, France). (A) shows the total content of real ambient PM whereas (B) provides a specific revelation of biological fraction of PM. DNA was colored with the SYTO® 9 fluorescent dye.

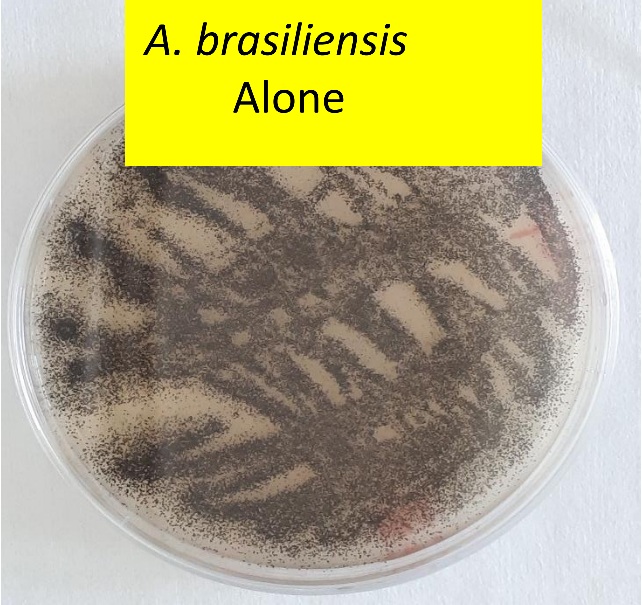

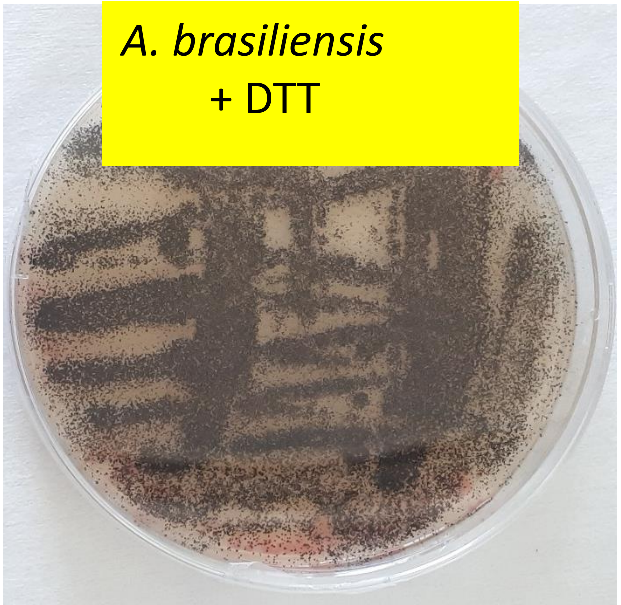

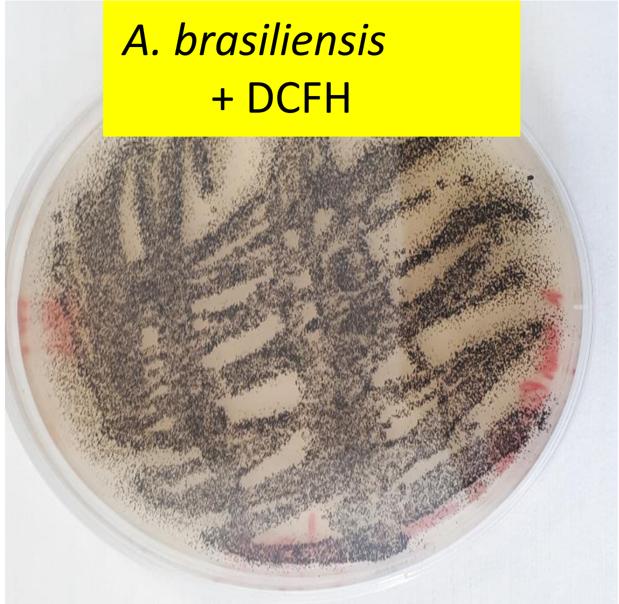

Identifiant	*A. brasiliensis* alone	*A. brasiliensis* + DTT	*A. brasiliensis* + DCFH
Spores/ml	$6{,}50*10^6$	$6{,}84*10^6$	$5{,}57*10^6$

Figure S 10A: Growth of *A. brasiliensis* spores incubated together or not with DTT/or DCFH onto Malt Agar culture medium (MA), concentration was obtained by gently scrapping spores into a sterile water resuspension followed by microscopic count.

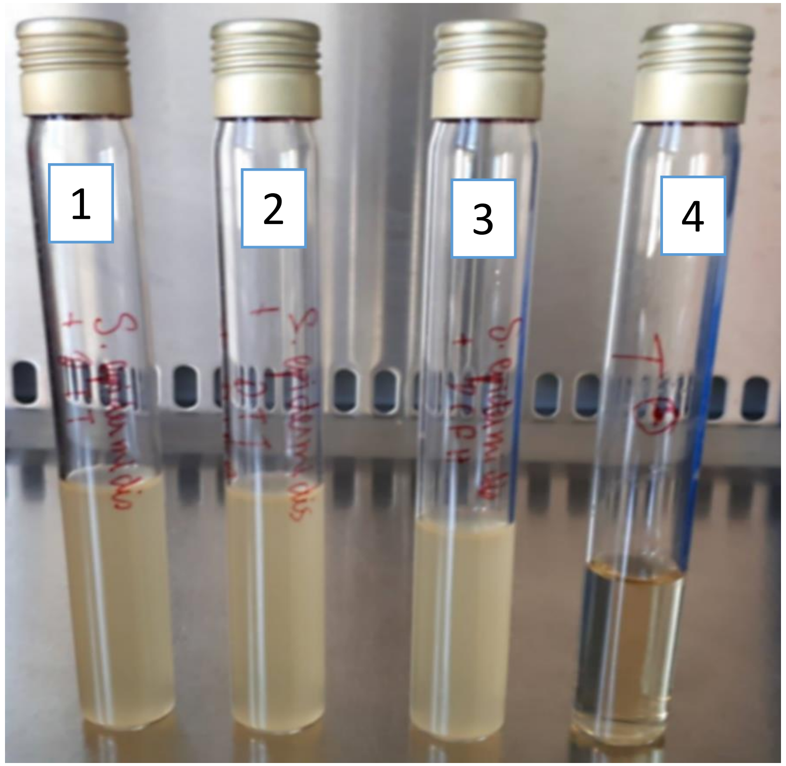

Identifiant	1	2	3	4
OD (550 nm)	1,283	1,305	1,422	0

Figure S 10B: Growth of *S. epidermidis* cells incubated together or with DTT/or DTT+DTNB/ or DCFH into Luria Bertani liquid (LB) culture medium: (1) *S. epidermidis* +DTT; (2) *S. epidermidis* +DTT+ DTNB; (3) *S. epidermidis* +DCFH-DA and (4) Negative control (LB without any cells). The Growth rate was determined by spectrophotometry (optical density (OD)) at 550 nm.

References used in the supplemental information.

1. Rattanavaraha, W. *et al.* The reactive oxidant potential of different types of aged atmospheric particles: An outdoor chamber study. *Atmos. Environ.* **45,** 3848–3855 (2011).
